# Plasma fatty acids and risk of colon and rectal cancers in the Singapore Chinese Health Study

**DOI:** 10.1038/s41698-017-0040-z

**Published:** 2017-11-23

**Authors:** Lesley M. Butler, Jian-Min Yuan, Joyce Yongxu Huang, Jin Su, Renwei Wang, Woon-Puay Koh, Choon-Nam Ong

**Affiliations:** 1 Cancer Control and Population Sciences, UPMC Hillman Cancer Center, University of Pittsburgh, Pittsburgh, PA USA; 2 Department of Epidemiology, Graduate School of Public Health, University of Pittsburgh, Pittsburgh, PA USA; 3 NUS Environmental Research Institute (NERI), National University of Singapore, Singapore, Singapore; 4 Duke-NUS Graduate Medical School Singapore, Singapore, Singapore; 5 Saw Swee Hock School of Public Health, National University of Singapore, Singapore, Singapore

## Abstract

Fatty acid composition in plasma captures both dietary intake and endogenous synthesis. Prospective analyses of plasma fatty acid composition are needed to establish the role of monounsaturated fatty acids (MUFAs) and polyunsaturated fatty acids (PUFAs) on risk of developing colorectal cancer. To evaluate associations between plasma fatty acid composition and colon or rectal cancer risk separately, a nested case-control study of 350 colorectal (211 colon and 139 rectal) cancer cases and an equal number of individually matched control subjects was conducted within the Singapore Chinese Health Study, a cohort of 63,257 men and women recruited between 1993 and 1998. Fatty acids in pre-diagnostic plasma were quantified using gas chromatography–tandem mass spectrometry. Conditional odds ratios (ORs) and 95% confidence intervals (CIs) comparing highest to lowest quartiles are presented. For colon cancer, inverse associations were reported with higher essential PUFAs, α-linolenic acid (OR = 0.41; 95% CI: 0.23, 0.73; *P*_trend_ = 0.005) and linoleic acid (OR = 0.43; 95% CI: 0.23, 0.82; *P*_trend_ = 0.008). Higher desaturase activity in the n-6 PUFA synthesis pathway estimated by the arachidonic:linoleic acid ratio was associated with increased colon cancer risk (OR = 3.53; 95% CI: 1.82, 6.85; *P*_trend_ = 0.006), whereas higher desaturase activity in the MUFA synthesis pathway estimated by the oleic:stearic acid ratio was associated with decreased colon cancer risk (OR = 0.42; 95% CI: 0.19, 0.92; *P*_trend_ = 0.024). There was no significant association between the essential fatty acids or the desaturase indices and rectal cancer risk. Endogenous synthesis of arachidonic and oleic acids has an impact on colon cancer development.

## Colorectal cancer: Dietary fat intake shapes colon cancer risk

Higher consumption of “good fats,” or their natural creation by the body, may help protect people from colon cancer. Jian-Min Yuan from the University of Pittsburgh, USA, and colleagues used samples from the Singapore Chinese Health Study to examine fatty acids contained in blood plasma from 350 people who later developed colorectal cancer and an equal number of matched control individuals. They found that higher blood levels—which reflect dietary intake—of the essential polyunsaturated fatty acids linolenic acid and α-linolenic acid were associated with a lower risk of colon cancer. So was oleic acid, a monounsaturated fat created by the body, whereas conversion of linolenic acid to another fatty acid called arachidonic acid boosted colon cancer risk. No effect was seen on rectal cancer risk. Diets rich in linolenic acid may help prevent colon cancer.

## Introduction

Colorectal cancer is the fourth most common cancer in the US.^1^ Worldwide, a trend of increasing incidence is observed among recently developed countries.^2^ Historically, Singapore had low-incidence rates of colorectal cancer, but most recent rates (33.3 per 100,000 from 2008 to 2012) have nearly reached those observed among Asians in the US.^1,3^ While it is undisputed that diet is an important contributor to colorectal cancer risk, the specific foods and nutrients that can be translated for prevention remain elusive.^4^

Fatty acids may contribute to colorectal carcinogenesis through a variety of mechanisms including the modulation of immunity, inflammation, and cell signaling.^5–7^ There is a large body of evidence supporting the role of lipid metabolism, particularly the effects of various eicosanoids generated from the cyclooxygenase and lipoxygenase metabolisms of arachidonic acid, an n-6 polyunsaturated fatty acid (PUFA), in the development and progression of colorectal cancer.^8^ Epidemiologic studies have, for the most part, relied on self-reported recall of usual diet to evaluate potential dietary fat–colorectal cancer associations, with only limited evidence for a positive association with animal fats.^9^ Prospective studies with comprehensive assessment of fatty acids using objective biomarkers that reflect the in vivo exposure are required to clarify their role in colorectal carcinogenesis. Until recently, only two nested case-control studies with relatively small sample size evaluated levels of fatty acids in pre-diagnostic blood samples in relation to colorectal cancer risk.^10,11^ Both studies reported statistically significant inverse associations with n-3 PUFAs, and no association with n-6 PUFAs, including arachidonic acid, or other long-chain fatty acids. In contrast, recent findings from a larger case-cohort study included a statistically significant positive association between plasma saturated fatty acids (SFAs) and colorectal cancer, and no association with n-3 PUFAs.^12^

**Fig. 1:**
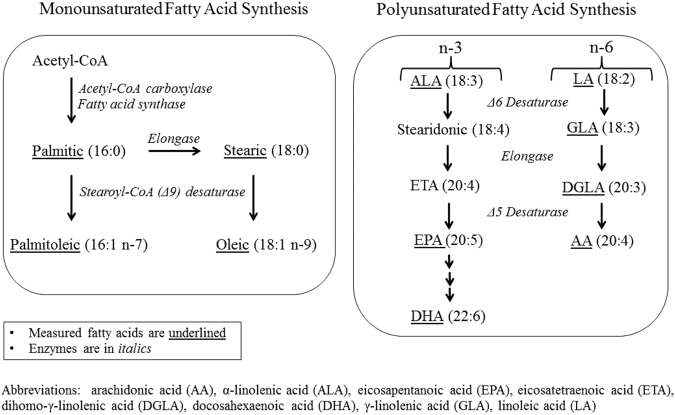
Synthesis pathways for omega (n)-7 and n-9 monounsaturated fatty acids and n-3 and n-6 polysaturated fatty acids. AA arachidonic acid, ALA α-linolenic acid, EPA eicosapentanoic acid, ETA eicosatetraenoic acid, DGLA dihomo-γ-linolenic acid, DHA docosahxaenoic acid, GLA γ-linolenic acid, LA linoleic acid

Arachidonic acid is primarily derived endogenously from the Δ5 and Δ6 desaturization and elongation of the essential fatty acid, linoleic (Fig. 1). On the other hand, Δ9 or stearoyl-coenzyme A desaturase-1 (SCD-1) plays an important role in the synthesis of monounsaturated fatty acids (MUFAs) from SFAs. The product-to-precursor ratios, such as arachidonic:linoleic acid ratio and oleic:stearic acid ratio may represent indices of hepatic Δ5 and Δ6 desaturase activity, and SCD-1 activity, respectively.^13,14^ There have been no prospective studies evaluating the association between these desaturase indices and risk of colorectal cancer. Utilizing the resources of the Singapore Chinese Health Study, we measured individual fatty acids in pre-diagnostic blood samples of 350 colorectal (211 colon and 139 rectal) cancer cases and individually matched control subjects using gas chromatography–tandem mass spectrometry (GC–MS/MS) to evaluate whether individual fatty acids and their ratios as estimates of desaturase activity are associated with colon and rectal cancers risk.

## Results

For colorectal cancer patients, the time from blood draw to cancer diagnosis ranged from <1 year to 12 years with a median of 3.3 years (interquartile range = 1.6–4.7). A wide range and low median was the results of only 3 of the 350 colorectal cancer cases whose blood samples were collected between 1994 and 1999 (an early protocol requesting blood collection from only 3% of early cohort participants). Regular NSAID use, defined as two or more times per week for 1 month or longer in the past year, was associated with reduced risk of colon cancer (Table 1). All other selected demographic or lifestyle factors were not associated with colon or rectal cancer (Table 1). Among control subjects, correlation coefficients (*r*) of plasma fatty acids ranged from 0.15 to 0.94 (Supplementary Table 1, Online Resource). Correlations with dietary levels were only present for plasma n-3 PUFA eicosapentanoic (EPA) and docosahexaenoic (DHA) acids, and essential α-linolenic and linoleic acids (*r* = 0.13–0.26) (Supplementary Table 2, Online Resource). Colon cancer patients had statistically significant lower mean concentrations of oleic, α-linolenic, linoleic, and γ-linolenic acids, and higher mean levels of the arachidonic:dihomo-γ-linolenic acid ratio (for Δ5 desaturase activity) and the arachidonic:linoleic acid ratio (for total n-6 PUFA desaturase activity) than control subjects (Table 2). Rectal cancer patients had comparable plasma levels of these fatty acids and desaturase indices to their control subjects.

**Table 1:** Selected demographic and lifestyle characteristics in patients with colon or rectal cancer and their matched control subjects, the Singapore Chinese Health Study

	Colon				Rectal			
	Cases	Controls	Odds ratio (95% CI)^a^	*P*_trend_	Cases	Controls	Odds ratio (95% CI)^a^	*P*_trend_
Number of subjects	211	211			139	139		
Age, mean (SD)^b^	59.9 (7.6)	59.9 (7.3)	–	–	59.4 (8.0)	59.2 (8.0)	–	–
Sex^b^								
Men	118	118	–	–	88	88	–	–
Women	93	93	–	–	51	51	–	–
Level of education								
No formal	50	44	1.00 (Referent)		35	31	1.00 (Referent)	
Primary	107	100	0.89 (0.54, 1.48)		67	62	0.91 (0.46, 1.81)	
Secondary or higher	54	67	0.70 (0.39, 1.24)	0.179	37	46	0.62 (0.28, 1.36)	0.207
Body mass index, kg/m^2^								
<20	34	34	1.00 (Referent)		22	18	1.00 (Referent)	
20–<24	103	118	0.86 (0.50, 1.46)		73	73	0.81 (0.40, 1.64)	
24–<28	53	47	1.13 (0.61, 2.10)		36	37	0.79 (0.36, 1.72)	
≥28	21	12	1.70 (0.75, 3.82)	0.159	8	11	0.58 (0.19, 1.80)	0.390
Cigarette smoking^c^								
Never	136	136	1.00 (Referent)		74	79	1.00 (Referent)	
Light	65	65	1.00 (0.65, 1.54)		57	54	1.20 (0.68, 2.09)	
Heavy	10	10	1.00 (0.41, 2.45)	1.00	8	6	1.52 (0.49, 4.71)	0.414
Alcohol, # drinks/week								
Nondrinker	165	166	1.00 (Referent)		109	115	1.00 (Referent)	
<7	34	37	0.93 (0.55, 1.57)		21	19	1.17 (0.60, 2.31)	
≥7	12	8	1.55 (0.60, 4.02)	0.638	9	5	2.06 (0.62, 6.87)	0.249
Physical activity, h/week								
0	142	141	1.00 (Referent)		90	88	1.00 (Referent)	
0.5–<4	38	40	0.94 (0.56, 1.58)		24	30	0.80 (0.43, 1.49)	
≥4	31	30	1.02 (0.58, 1.80)	1.00	25	21	1.12 (0.58, 2.19)	0.869
Diabetes								
No	186	190	1.00 (Referent)		120	130	1.00 (Referent)	
Yes	25	21	1.22 (0.66, 2.28)	0.528	19	9	2.11 (0.96, 4.67)	0.065
Regular NSAID use^d^								
Never	193	182	1.00 (Referent)		119	123	1.00 (Referent)	
Ever	10	21	0.39 (0.16, 0.93)	0.034	17	13	1.36 (0.63, 2.97)	0.435

*CI* confidence interval, *NSAID* nonsteroidal anti-inflammatory drug

^a^ Odds ratios were calculated from conditional logistic regression models

^b^ Age and sex were matching factors, thus the odds ratios and *P* values were not calculated

^c^ Heavy smokers were those who started to smoke cigarettes before 15 years of age and smoked at least 13 cigarettes per day. Light smokers were those who started to smoke cigarettes at or after 15 years of age or smoked 12 or less cigarettes per day

^d^ NSAID use was collected after baseline at the first follow-up questionnaire. Regular use was defined as two or more times per week for 1 month or longer

**Table 2:** Geometric means of plasma fatty acids and their desaturase indices among patients with colon or rectal cancer and their matched control subjects, the Singapore Chinese Health Study

	Colon			Rectal		
	Cases (*n* = 211)	Controls (*N* = 211)	*P*-value^a^	Cases (n = 139)	Controls (*N* = 139)	*P-* value^a^
Monounsaturated fatty acid (MUFA) synthesis pathway						
Saturated fatty acids						
Palmitic acid (16:0), µmol/dL	265.82	282.64	0.078	277.74	269.14	0.415
Stearic acid (18:0), µmol/dL	92.62	97.32	0.097	96.74	93.88	0.369
MUFAs						
Palmitoleic acid (16:1), µmol/dL	18.54	20.82	0.061	20.88	20.40	0.765
Oleic acid (18:1), µmol/dL	282.76	311.10	0.020	310.14	293.14	0.236
Stearoyl-coenzyme A desaturase (SCD)-1 indices						
Palmitoleic:Palmitic acid ratio	0.06	0.08	0.221	0.08	0.08	0.881
Oleic:Stearic acid ratio	3.48	3.64	0.055	3.64	3.54	0.362
n-3 Polyunsaturated fatty acids (PUFAs)						
α-Linolenic acid (18:3), µmol/dL	2.50	2.88	0.009	2.78	2.58	0.260
Eicosapentanoic acid (20:5), µmol/dL	3.52	3.48	0.862	3.48	3.52	0.873
Docosahexaenoic acid (22:6), µmol/dL	24.04	23.68	0.777	25.02	25.82	0.604
n-6 PUFA synthesis pathway						
n-6 PUFAs						
Linoleic acid (18:2) (LA), µmol/dL	569.04	600.26	0.044	591.76	570.82	0.286
γ-Linolenic acid (18:3) (GLA), µmol/dL	1.54	1.82	0.030	1.58	1.50	0.590
Dihomo-γ-linolenic acid (20:3) (DGLA), µmol/dL	7.38	8.06	0.064	7.88	7.20	0.154
Arachidonic acid (20:4) (AA), µmol/dL	73.16	69.72	0.207	75.80	71.38	0.210
n-6 PUFA desaturase indices (DI)						
GLA:LA ratio (for Δ6 DI) (×1000)	2.7	3.2	0.137	2.6	2.6	0.869
AA:DGLA ratio (for Δ5 DI)	9.84	8.58	0.002	9.56	9.86	0.525
AA:LA ratio (for total n-6 PUFA DI) (×1000)	129	116	0.005	128	125	0.615

^a^ *P*-value for difference between case and control groups were derived from one-way ANOVA for log-transformed measures of plasma fatty acids

The associations with risk of colon and rectal cancers are presented for plasma fatty acids or desaturase indices involved in the MUFA synthesis pathway (Table 3) and in the PUFA synthesis pathway (Table 4). The corresponding associations with colorectal cancer risk are shown in Supplementary Table 3. Statistically significant inverse associations with colon cancer risk were observed for the SFA palmitic acid and the MUFA oleic acid (Table 3). The inverse association between palmitic acid and colon cancer risk was completely explained by MUFAs; the plasma total MUFA-adjusted odds ratios (ORs) (95% confidence intervals (CIs)) for the 2nd, 3rd, and 4th quartile of palmitic acid were 0.87 (0.44, 1.72), 0.93 (0.44, 1.95), and 0.85 (0.26, 2.72), respectively (*P*_trend_ = 0.828). On the other hand, adjustment for plasma SFAs did not fully explain the inverse oleic acid–colon cancer risk association; the plasma total SFA-adjusted ORs (95% CIs) for the 2nd, 3rd, and 4th quartile of oleic acid were 0.58 (0.31, 1.09), 0.76 (0.37, 1.53), 0.49 (0.17, 1.43), respectively (*P*_trend_ = 0.306). The oleic:stearic acid ratio was significantly associated with reduced risk of colon cancer. The individual SFAs and MUFAs and their ratios were not associated with rectal cancer risk (Table 3).

**Table 3:** Adjusted odds ratios (95% confidence intervals)  of colon and rectal cancer by quartile levels of plasma saturated and monounsaturated fatty acids (MUFA) and their desaturase indices, the Singapore Chinse Health Study^a^

	1st (low)	2nd	3rd	4th (high)	*P*_trend_
Colon cancer					
Saturated fatty acids					
Palmitic acid (16:0)	1.00 (Referent)	0.77 (0.40–1.49)	0.71 (0.37–1.36)	0.41 (0.20–0.83)	0.016
Stearic acid (18:0)	1.00 (Referent)	0.97 (0.55–1.69)	0.96 (0.56–1.64)	0.55 (0.30–0.99)	0.070
MUFAs					
Palmitoleic acid (16:1)	1.00 (Referent)	0.79 (0.44–1.42)	0.81 (0.46–1.41)	0.51 (0.28–0.95)	0.051
Oleic acid (18:1)	1.00 (Referent)	0.56 (0.30–1.04)	0.69 (0.37–1.28)	0.39 (0.20–0.76)	0.015
Stearoyl-coenzyme A desaturase (SCD)-1 indices					
Palmitoleic:Palmitic acid ratio	1.00 (Referent)	0.79 (0.43–1.44)	0.93 (0.54–1.59)	0.58 (0.31–1.08)	0.165
Oleic:Stearic acid ratio	1.00 (Referent)	0.79 (0.41–1.51)	0.58 (0.28–1.23)	0.42 (0.19–0.92)	0.024
Rectal cancer					
Saturated fatty acids					
Palmitic acid (16:0)	1.00 (Referent)	0.92 (0.46–1.83)	1.15 (0.55–2.44)	1.50 (0.65–3.44)	0.312
Stearic acid (18:0)	1.00 (Referent)	1.55 (0.78–3.09)	0.84 (0.39–1.82)	1.72 (0.79–3.75)	0.356
MUFAs					
Palmitoleic acid (16:1)	1.00 (Referent)	0.81 (0.40–1.62)	0.98 (0.50–1.90)	1.10 (0.51–2.39)	0.768
Oleic acid (18:1)	1.00 (Referent)	1.70 (0.86–3.36)	1.43 (0.68–3.01)	1.77 (0.80–3.91)	0.219
SCD-1 indices					
Palmitoleic:Palmitic acid ratio	1.00 (Referent)	0.90 (0.45–1.80)	1.12 (0.49–2.58)	1.09 (0.50–2.36)	0.743
Oleic:Stearic acid ratio	1.00 (Referent)	1.54 (0.76–3.12)	1.26 (0.62–2.58)	1.15 (0.48–2.76)	0.730

^a^ Odds ratios are adjusted for body mass index (<20, 20–24, 24–28, ≥28 kg/m^2^), smoking (never, light, heavy), education level (none, primary, ≥secondary), alcohol use (none, <7, ≥7 drinks/week), weekly physical activity (yes, no), history of diabetes (yes, no), and use of nonsteroidal anti-inflammatory drugs (NSAIDs) (yes, no)

**Table 4:** Adjusted odds ratios (95% confidence intervals) of colon and rectal cancers by quartile levels of plasma polyunsaturated fatty acids (PUFA) and desaturase indices in n-6 PUFA) synthesis pathway, The Singapore Chinese Health Study^a^

	1st (low)	2nd	3rd	4th (high)	*P*_trend_
Colon cancer					
n-3 PUFAs					
α-Linolenic acid (18:3)	1.00 (Referent)	0.56 (0.32–1.00)	0.64 (0.37–1.11)	0.41 (0.23–0.73)	0.005
Eicosapentanoic acid (20:5)	1.00 (Referent)	1.06 (0.60–1.86)	1.18 (0.69–2.02)	0.93 (0.54–1.62)	0.931
Docosahexaenoic acid (22:6)	1.00 (Referent)	1.47 (0.83–2.62)	1.32 (0.74–2.34)	1.11 (0.59–2.07)	0.855
n-6 PUFAs					
Linoleic acid (18:2) (LA)	1.00 (Referent)	0.63 (0.35–1.13)	0.52 (0.29–0.93)	0.43 (0.23–0.82)	0.008
γ-Linolenic acid (18:3) (GLA)	1.00 (Referent)	0.62 (0.33–1.15)	0.68 (0.36–1.27)	0.54 (0.30–1.00)	0.085
Dihomo-γ-linolenic acid (20:3) (DGLA)	1.00 (Referent)	0.81 (0.45–1.43)	0.80 (0.44–1.46)	0.54 (0.29–1.00)	0.060
Arachidonic acid (20:4) (AA)	1.00 (Referent)	1.36 (0.78–2.37)	2.18 (1.16–4.08)	1.52 (0.82–2.81)	0.111
n-6 PUFA desaturase indices (DI)					
GLA:LA ratio (for Δ6 DI)	1.00 (Referent)	1.09 (0.60–1.98)	1.19 (0.66–2.13)	0.61 (0.33–1.10)	0.140
AA:DGLA ratio (for Δ5 DI)	1.00 (Referent)	1.28 (0.70–2.31)	2.77 (1.53–5.03)	2.51 (1.39–4.53)	<0.001
AA:LA ratio (for total n-6 PUFA DI)	1.00 (Referent)	2.90 (1.52–5.55)	1.28 (0.69–2.37)	3.53 (1.82–6.85)	0.006
Rectal cancer					
n-3 PUFAs					
α-Linolenic acid (18:3)	1.00 (Referent)	1.00 (0.50–2.03)	1.38 (0.66–2.91)	1.70 (0.84–3.43)	0.087
Eicosapentanoic acid (20:5)	1.00 (Referent)	1.07 (0.52–2.20)	0.64 (0.31–.35)	0.89 (0.42–1.91)	0.516
Docosahexaenoic acid (22:6)	1.00 (Referent)	0.79 (0.38–1.66)	0.63 (0.30–1.31)	0.72 (0.32–1.66)	0.349
n-6 PUFAs					
LA (18:2)	1.00 (Referent)	0.88 (0.44–1.75)	1.64 (0.78–3.44)	1.15 (0.51–2.58)	0.394
GLA (18:3)	1.00 (Referent)	1.43 (0.65–3.14)	1.43 (0.72–2.84)	1.59 (0.75–3.34)	0.232
DGLA (20:3)	1.00 (Referent)	0.76 (0.39–1.51)	0.94 (0.44–2.01)	2.36 (1.02–5.44)	0.068
AA (20:4)	1.00 (Referent)	0.86 (0.40–1.82)	1.93 (0.86–4.31)	1.44 (0.63–3.27)	0.200
n-6 PUFA DIs					
GLA:LA ratio (for Δ6 DI)	1.00 (Referent)	1.14 (0.56–2.31)	1.05 (0.54–2.03)	1.47 (0.65–3.34)	0.476
AA:DGLA ratio (for Δ5 DI)	1.00 (Referent)	0.66 (0.31–1.37)	0.56 (0.26–1.21)	0.68 (0.33–1.42)	0.366
AA:LA ratio (for total n-6 PUFA DI)	1.00 (Referent)	1.24 (0.57–2.72)	1.98 (0.88–4.46)	1.08 (0.50–2.32)	0.711

^a^ Odds ratios are adjusted for body mass index (<20, 20–24, 24–28, ≥28 kg/m^2^), smoking (never, light, heavy), education level (none, primary, ≥secondary), alcohol use (none, <7, ≥7 drinks/week), weekly physical activity (yes, no), history of diabetes (yes, no), and use of nonsteroidal anti-inflammatory drugs (NSAIDs) (yes, no)

Statistically significant inverse associations with colon cancer risk were observed for the essential PUFAs α-linolenic and linoleic acids (Table 4). The positive arachidonic acid–colon cancer risk association was stronger and the trend became statistically significant among individuals without a history of regular NSAID use; ORs (95% CIs) for colon cancer for 2nd, 3rd, 4th quartile were 1.46 (0.80, 2.67), 2.18 (1.10, 4.34), and 1.96 (1.00, 3.84), respectively, (*P*_trend_ = 0.032). In addition, higher levels of both arachidonic:dihomo-γ-linolenic acid ratio and arachidonic:linoleic acid ratio were associated with statistically significant increased risk of colon cancer (Table 4). The individual PUFAs and desaturase indices were not significantly associated with rectal cancer risk, except for a positive association with dihomo-γ-linolenic acid (Table 4).

In sensitivity analyses, we evaluated whether fasting status had any impact on the associations between plasma fatty acids and risk of colon and rectal cancers. The associations with colon cancer risk was stronger for fatty acids assessed in nonfasting state than in fasting state, although none of their difference was statistically significant (Supplementary Table 4, Online Resource). We also examined the association between plasma fatty acid levels and colon cancer risk after excluding cases that were diagnosed within first 2 years or first 4 years after blood draw. In general, associations were attenuated and no longer statistically significant, with the following exceptions. A stronger association was observed for the oleic:stearic acid ratio after excluding the cases and matched controls from the first 2 years (OR = 0.27; 95% CI: 0.09, 0.78; *P*_trend_ = 0.02, for fourth vs. first quartile) (Supplementary Table 5, Online Resource). After excluding cases and matched controls from the first 4 years, stronger associations were observed for arachidonic acid and the arachidonic:linoleic acid ratio. The odds ratios (95% CIs) for fourth vs. first quartile were 3.96 (1.40, 11.21; *P*_trend_ < 0.01) and 11.97 (2.93, 48.87; *P*_trend_ = 0.01), respectively (Supplementary Table 5, Online Resource). Statistically significant heterogeneity according to follow-up time was only present for arachidonic acid (*P*_heterogeneity_ = 0.03) (Supplementary Table 6, Online Resource).

## Discussion

Our main findings included statistically significant inverse associations with colon cancer risk for higher levels of the essential PUFAs α-linolenic and linoleic acids, the major contributing MUFA oleic acid, and SCD-1 index reflecting increased synthesis of oleic acid. Our results also included a statistically significant positive association with colon cancer for the desaturase indices reflecting increased synthesis of arachidonic acid.

Plasma essential fatty acids reflect dietary intake. The primary dietary sources for α-linolenic acid are flaxseed, walnuts, and canola and soybean oils; and for linoleic acid, soybean, corn, and safflower oils, as well as nuts (i.e., pine nuts, pecans, brazil nuts), and sunflower seeds. The preventive effects of α-linolenic acid are generally considered to be due to its role as a precursor for the biosynthesis of EPA and DHA. However, this conversion is extremely low.^15^ In a human feeding study, dietary α-linolenic acid had little impact on plasma EPA or DHA.^16^ We did not report associations with EPA or DHA and colorectal cancer risk. Thus, our finding supports an alternative role of the essential n-3 PUFA α-linolenic acid against the development of colon cancer by reducing inflammation^17,18^ and inhibiting proliferation and invasion,^19^ rather than as a precursor. In addition, our finding supports a potential preventive role for the essential n-6 PUFA linoleic acid in colon carcinogenesis that may be due in part to its effect on increasing apoptosis and decreasing cancer cell proliferation.^20^

The present study showed a positive association between plasma level of arachidonic acid and colon cancer risk, especially among individuals without regular use of nonsteroidal anti-inflammatory drugs (NSAIDs). Furthermore, the product-to-precursor ratios of arachidonic to dihomo-γ-linolenic or linoleic acids as estimates of the enzymatic activity for the endogenous synthesis of arachidonic acid were strongly associated with increased risk of developing colon cancer. The major source of plasma arachidonic acid is from desaturization and elongation of linoleic acid. The role of arachidonic acid in colon cancer development is as the precursor of proinflammatory eicosanoids (e.g., two-series prostacyclins and thromboxanes, and four-series leukotrienes).^21,22^ These arachidonic acid metabolites are established promoters of colon carcinogenesis.^23^ Inhibition of the conversion of arachidonic acid to prostaglandins by inhibiting cyclooxygenases is the mechanism underlying the protective effects of NSAID use on colorectal cancer risk.^24,25^

There have been no prospective studies reporting on the estimated n-6 PUFA desaturase activity and colon cancer risk. One study reported a statistically significant positive association for Δ5 desaturase index with risk of total cancer.^26^ The other reported a statistically significant, positive relationship for Δ5 desaturase index with plasma C-reactive protein, a risk factor for colorectal cancer.^27,28^ Experimental studies showed that the inhibition of synthesis pathway of arachidonic acid resulted in significantly reduced number and size of intestinal tumors along with the significantly increased linoleic and decreased arachidonic acids in tissue phospholipids.^29^ Our findings with those of animal^29–31^ and human studies^26,27^ support the hypothesis that increased capacity to synthesize arachidonic acid contributes to the development of colon cancer.

We reported a statistically significant 77% decrease in colon cancer risk with highest quartile of the oleic:stearic acid ratio, reflecting increased SCD-1 activity. SCD-1 is a key regulator in lipid metabolism and controls the homeostasis of MUFAs and SFAs.^32^ Cancer cells demand higher than normal levels of lipid biosynthesis, particularly de novo synthesis of MUFAs to support the metabolic transformation that lead to their rapid growth.^33^ Thus, SCD-1 is found to be overexpressed in human malignant tissues, including human colon tumor tissue.^34^ SCD-1, however, has a dual role in that it can also suppress cellular inflammation and stress responses in a variety of cell types and disease conditions.^35,36^ For example, decreased SCD-1 activity is associated with proinflammatory activity and worse disease severity in a mouse model of inflammatory bowel disease.^37^ Patients with active ulcerative colitis, a type of inflammatory bowel disease associated with increased colorectal cancer risk, had lower *SCD1* gene expression compared to expression levels among healthy controls (*P* = 0.045).^38^ Our finding for an inverse association with the oleic:stearic acid ratio, especially for individuals with longer time interval (i.e., >2 years prior to cancer diagnosis) between specimen collection and cancer diagnosis is consistent with the beneficial effects of SCD-1 on colorectal cancer development.

In the present study, we did not find any association between fatty acids and rectal cancer risk. No previous study has evaluated the association between biomarkers of fatty acids and rectal cancer risk. Our study is consistent with three prospective cohort studies in Japanese,^39^ Chinese,^40^ and Swedish^41^ populations that studied the associations between dietary fatty acids and rectal cancer risk and found no dose–risk trend. Given that all previous three studies and the current study have a relatively small number of rectal cancer cases, studies with a larger sample sizes providing a greater statistical power are warranted to clarify the associations between fatty acids and rectal cancer risk.

Results from prospective analyses of circulating fatty acids in relation to colorectal cancer risk have been reported from three studies.^10–12^ The first nested case-control study reported statistically significant inverse associations with colorectal cancer among Japanese men (83 cases and 241 controls) for serum n-3 PUFAs α-linolenic and docosapentaenoic acids.^11^ The second study was conducted among a US population of men who participated in a randomized controlled trial of aspirin use.^10^ There were no associations among total subjects. Among men not assigned to aspirin (92 cases and 142 controls), a statistically significant inverse association with total n-3 PUFAs in blood for colorectal cancer risk was reported. The third study was conducted using a case-cohort design and included 395 colorectal cancer cases identified from a prospective cohort in Australia.^12^ A statistically significant positive association was reported with total SFAs, and no association with n-3 PUFAs or n-6 PUFAs for colorectal cancer. In summary, results from two^10,11^ of the three previous studies support an inverse association with n-3 PUFAs blood levels, and only one study reported a positive association with plasma SFAs.^12^ In our study, we reported a statistically significant, inverse association for the n-3 PUFA α-linolenic acid and colon cancer risk among men and women. Thus, the findings for circulating n-3 PUFAs and colorectal cancer are consistent across three of the four studies.

Reasons for the inconsistent findings across studies could include the different methods used to measure fatty acids in blood [i.e., gas chromatography followed by flame ionization detection (FID) vs. mass spectrometry], as well as differences in study design (i.e., nested case-control vs. case-cohort). In general, mass spectrometry has greater specificity than the FID method used in the previous studies. It is possible that misclassification due to batch effects may impact results from a case-cohort study, if the fatty acids were not measured among the cases and the sub-cohort at the same time, unless this was taken into account in the statistical analyses.

Strengths of the present study included the measurement of the major fatty acids in both MUFA and PUFA synthesis pathways, thus allowing for the examination of the effects of fatty acid composition, as well as endogenous synthesis capacity on risk of colorectal cancer. Another strength was the prospective study design with biospecimen samples that were collected prior to cancer diagnosis, thus minimizing the potential for biased biomarker levels in cancer patients due to the subclinical symptoms and progression of underlying disease. Except for the arachidonic acid–colon cancer association, there was no evidence for heterogeneity according to time from blood draw to colon cancer diagnosis (all *P*_heterogeneity_ values > 0.2). The relatively large sample sizes of both colon (211 cases) and rectal (139 cases) allowed us to examine the effect of these fatty acids and their synthesis pathways on the risk of colon and rectal cancers separately, given that several studies reported that the risk profiles for colon cancer may be different from those of rectal cancer.^42–44^ Our study is the first to demonstrate that risk associations with plasma fatty acids and their desaturase indices for colon cancer are different from those for rectal cancer. One major limitation of the present study was a one-time assessment of fatty acid levels in plasma, which may not represent their long-term levels of exposure due to likely changes in diet and lifestyles. It is conceivable that measurement errors from the one-time assessment could occur nondifferentially in both cancer patients and control subjects, which would attenuate the observed exposure–disease risk associations towards the null. Our results may also be limited because it was not feasible to measure actual desaturase activity in our study participants. Instead, we used the well-established approach of serum fatty acid product-to-precursor ratios as estimates of desaturase activity.^14^ Caution should be taken in interpreting the associations identified from the product-to-precursor ratios as estimates of desaturase activity, because it assumes that only the enzyme influences the ratio levels, when in fact other factors, such as genetic variation and reactions upstream and downstream from the target reaction are likely to influence fatty acid levels.^45^

In conclusion, the present study demonstrated an inverse association with essential PUFAs α-linolenic and linoleic acids, and the major contributing MUFA oleic acid, and its increased synthesis capacity with colon cancer risk. The present study also showed a positive association with high plasma levels of arachidonic acid and its increased synthesis capacity on colon cancer risk. These novel findings, if confirmed, have implications for colon cancer prevention.

## Methods

### Study population

The design of the Singapore Chinese Health Study has been previously described.^46^ Eligible subjects were permanent residents or citizens of Singapore aged 45–74 years and belonging to one of the two major Chinese dialect groups (Cantonese and Hokkien). At baseline between 1993 and 1998, all cohort members completed an in-person interview that included a validated 165-item food frequency questionnaire.^47^ The questionnaire also elicited information on demographics, current physical activity and medical history. A follow-up questionnaire was conducted between 1999 and 2004 among 83% of the original cohort, and obtained information about use of NSAIDs.

Biospecimens were collected from a 3% random sample of the entire cohort between 1994 and 1999 according to an early study protocol. Beginning in 2000, the study protocol requested the collection of biospecimens to be extended to all consented participants of the entire cohort. By April 2005, biospecimens, including a nonfasting blood sample, were obtained from 32,543 subjects, representing a 60% consent rate. All blood components were separated immediately and stored at −80 °C until analyzed. Written, informed consent was obtained from all study participants. The study was approved by the Institutional Review Boards at the National University of Singapore and the University of Pittsburgh.

### Identification of cancer cases and control subjects

Cancer diagnoses were identified by linkage analysis for all cohort members with the Singapore Cancer Registry.^48^ By November 1, 2008, 350 cohort participants with available pre-diagnostic blood samples developed incident colorectal (211 colon and 139 rectal) cancer and were included in this study. For each case, one control subject was randomly chosen among all eligible cohort participants who were alive and free of cancer at the date of diagnosis of the index case. The control subject was individually matched to an index case by sex, dialect group (Cantonese, Hokkien), age at baseline interview (within 3 years), date of baseline interview (within 2 years), and date of biospecimen collection (within 6 months). All 350 incident colorectal cancer cases available were included in the present study. Based on one control per case, the present study would have 80% statistical power to detect a minimal odds ratio of 1.5 for colorectal cancer.

### Laboratory methods

Two aliquots of plasma samples from a given matched case-control pair were plated next to each other in a random order and assayed for all fatty acids in the same laboratory batch. Laboratory personnel were blinded to the case/control status of the plasma aliquots. The separation and quantification of 11 fatty acids was achieved by GC–MS/MS, as previously described.^49^ In brief, 60 µL of type I internal standard (30 µg/mL 2-methylhexadecanoic acid in MeOH) was heated at 80 °C for 10 min after vigorous shaking. After cooling to 25 °C, 0.5 mL BF_3_–MeOH reagent (14%, w/v) was added and heated at 80 °C for 3 min. After cooling, 0.5 mL hexane and 0.2 mL NaCl saturated solution were added, then vortexed for 3 min and centrifuged for 5 min at 3000 rpm. Then 0.2 mL clear n-hexane top layer was transferred and dried under a stream of N_2,_ and then re-dissolved with 0.1 mL type II internal standard solution (0.5 µg/mL ethyl nonadecanoate in hexane). The fatty acid methyl esters were then analyzed with an Agilent gas chromatography (Model 7890, Shanghai, China) equipped with a 7000A QQQ triplequadrupole mass detector (CA, US) and an auto sample injector (Model 7860B, Shanghai, China). The range of limits of detection for all measured fatty acids was 0.039–0.156 µg/mL. The within-batch precision ranged from 1.50 to 8.18% and the between-batch precision ranged from 1.54 to 7.76%.^49^

### Statistical analysis

The distributions of plasma fatty acids (µmol/dL) were markedly skewed toward high values. Thus, statistical analyses were performed on logarithmically transformed values. The analysis of covariance (ANOVA) method was used to examine differences in mean concentrations of plasma fatty acids between cases and control subjects. The variances in fatty acid measurements in cases were comparable with those in controls. Desaturase indices were calculated using product-to-precursor ratios of individual fatty acids as follows (also see Fig. 1): Δ9 desaturase (or SCD-1) was estimated as palmitoleic (16:1): pentadecylic (16:0) acid ratio and oleic (18:1): stearic (18:0) acid ratio, Δ5 desaturase as arachidonic (20:4n-6):dihomo-γ-linolenic (20:3n-6) acid ratio, Δ6 desaturase as γ-linolenic (18:3n-6): linoleic (18:2n-6) acid ratio; and total n-6 PUFA desaturase activity as arachidonic (20:4n-6): linoleic (18:2n-6) acid ratio. These fatty acid ratios have been well established as indices of desaturase activity in humans.^27,50,51^ In contrast, the product-to-precursor ratios of n-3 PUFAs do not reflect the respective enzymatic activities, because of the substantially higher linoleic acid vs. α-linolenic acid level.

To calculate ORs and their corresponding 95% CIs and *P*-values we performed conditional logistic regression analyses. Study subjects were grouped into quartile categories based on the distributions of plasma fatty acids among controls (Supplement Table 7, Online Resource). To compute the linear trend tests we used ordinal variables, where each category was assigned the median value within each quartile of the corresponding fatty acid or desaturase activity index. To adjust for potential confounding effects, the regression models included the following variables: level of education (no formal education, primary school, secondary school or higher), body mass index (<20, 20–<24, 24–<28, ≥28 kg/m^2^), cigarette smoking (“heavy” = started to smoke before age 15 and smoked ≥13 cigarettes per day, “light” = all nonheavy smokers, or never smokers),^52^ alcohol consumption (nondrinker, <7 drinks/week, ≥7 drinks/week), any weekly physical activity (no, yes), and self-reported diabetes (no, yes).^53^ To evaluate whether time from blood draw to cancer diagnosis modified the observed associations between plasma fatty acids and colorectal cancer risk, we conducted stratified analyses by time period using the median time as the cutpoint (<3 and ≥3 years). In addition, to evaluate the opportunity for reverse causation, the main findings were re-assessed after removing cases (and their matched controls) diagnosed within 2 or 4 years following blood draw.

Statistical computing was conducted using the SAS version 9.3 statistical software package (SAS Institute Inc., NC). All *P-*values < 0.05 were considered statistically significant.

The data that support the findings of this study are available from University of Pittsburgh through the contact author. The data will be released upon approval of user’s agreement by University of Pittsburgh Office of Research.

Methods were performed in accordance with relevant regulations and guidelines.

## Electronic supplementary material

Supplementary Table 1

Supplementary Table 2

Supplementary Table 3

Supplementary Table 4

Supplementary Table 5

Supplementary Table 6

Supplementary Table 7

## Electronic supplementary material

**Supplementary information** accompanies the paper on the *npj Precision Oncology* website (10.1038/s41698-017-0040-z).
