# Supplementary material for: Plasma fatty acids and risk of colon and rectal cancers in the Singapore Chinese Health Study
Source: NPJ Precis Oncol. 2017 Nov 23;1:38. doi: 10.1038/s41698-017-0040-z (PMC5871823; doi:10.1038/s41698-017-0040-z)
Supplement: Supplementary file 1 — Supplementary Table 1 [file 41698_2017_40_MOESM1_ESM.docx]

**Plasma fatty acids and risk of colon and rectal cancers in the Singapore Chinese Health Study**

Lesley M. Butler^1,2^, Jian-Min Yuan^1,2^, Joyce Yongxu Huang^1,2^, Jin Su^3^, Renwei Wang^1^, Woon-Puay Koh^4,5^, Choon Nam Ong^3^

^1^ Cancer Control and Population Sciences, University of Pittsburgh Cancer Institute, Pittsburgh, Pennsylvania; ^2^ Department of Epidemiology, Graduate School of Public Health, University of Pittsburgh, Pittsburgh, Pennsylvania; ^3^ NUS Environmental Research Institute (NERI), National University of Singapore, Singapore; ^4^ Duke-NUS Graduate Medical School Singapore, Singapore; ^5^ Saw Swee Hock School of Public Health, National University of Singapore, Singapore

**Corresponding author:**

Jian-Min Yuan, PhD, UPMC Cancer Pavilion, Suite 4C, 5150 Centre Avenue, Pittsburgh, PA 15232; Telephone: (412) 864-7889; Fax: (412) 864-7838; E-mail: yuanj@upmc.edu

The supplemental data included number of cases and controls by quartile levels of biomarkers, correlation coefficients of biomarkers of fatty acids, and additional analysis for the associations between fatty acids and colorectal cancer risk.

**Supplementary Table 1.** Correlation coefficients between plasma fatty acids and desaturase indices (DI), among control subjects (n=316)

**Supplementary Table 2.** Spearman correlation coefficients between plasma fatty acids and dietary fatty acids, among control subjects (n=350)

**Supplementary Table 3.** Adjusted odds ratios and 95% confidence intervals of colorectal cancer by quartile levels of plasma fatty acids and desaturase indices in monounsaturated fatty acid (MUFA) and polyunsaturated (PUFA) synthesis pathways

**Supplmental Table 4.** Adjusted odds ratios and 95% confidence intervals of colon cancer by quartile levels of plasma palmitic acid (16:0), oleic acid (18:1), a-Linolenic acid (18:3), and AA:DGLA, a desaturase indice in n-6 polyunsaturated fatty acid (PUFA) synthesis pathway, stratified by fasting status

**Supplementary Table 5.** Adjusted odds ratios and 95% confidence intervals of colon cancer comparing highest to lowest quartile of selected plasma fatty acids and desaturase indices excluding cases identified during the first 2 and 4 years of follow-up and their matched controls.

**Supplementary Table 6.** Adjusted odds ratios and 95% confidence intervals of colon cancer comparing highest to lowest quartile of selected plasma fatty acids and desaturase indices excluding cases according to median follow-up time.

**Supplementary Table 7.** Number of colon and rectal cancer cases and their matched controls by quartile levels of plasma fatty acids and their desaturase indices

**Supplementary Table 1.** Spearman correlation coefficients between plasma fatty acids and desaturase indices (DI), among control subjects (n=316*)

|  | SFAs | | MUFAs | | SCD-1 indices | | n-3 PUFAs | | | n-6 PUFAs | | | | n-6 PUFA DIs | | |
| --- | --- | --- | --- | --- | --- | --- | --- | --- | --- | --- | --- | --- | --- | --- | --- | --- |
|  | PA | SA | PLA | OA | PLA/PA | OA/SA | ALA | EPA | DHA | LA | GLA | DGLA | AA | GLA/LA | AA/DGLA | AA/LA |
| SFAs |  |  |  |  |  |  |  |  |  |  |  |  |  |  |  |  |
| Palmitic acid (16:0) (PA) | 1.00 | **0.84^†^** | **0.68^†^** | **0.94^†^** | **0.23^†^** | **0.64^†^** | **0.59^†^** | **0.29^†^** | **0.55^†^** | **0.69^†^** | **0.41^†^** | **0.61^†^** | **0.50^†^** | **0.18^†^** | **-0.27^†^** | 0.01 |
| Stearic acid (18:0) (SA) |  | 1.00 | **0.64^†^** | **0.82^†^** | **0.28^†^** | **0.27^†^** | **0.61^†^** | **0.33^†^** | **0.55^†^** | **0.64^†^** | **0.50^†^** | **0.65^†^** | **0.55^†^** | **0.28^†^** | **-0.29^†^** | 0.10 |
| MUFAs |  |  |  |  |  |  |  |  |  |  |  |  |  |  |  |  |
| Palmitoleic acid (16:1) (PLA) |  |  | 1.00 | **0.68^†^** | **0.85^†^** | **0.41^†^** | **0.46^†^** | **0.15^†^** | **0.42^†^** | **0.39^†^** | **0.56^†^** | **0.72^†^** | **0.48^†^** | **0.43^†^** | **-0.44^†^** | **0.22^†^** |
| Oleic acid (18:1) (OA) |  |  |  | 1.00 | **0.27^†^** | **0.75^†^** | **0.59^†^** | **0.22^†^** | **0.49^†^** | **0.66^†^** | **0.39^†^** | **0.59^†^** | **0.46^†^** | **0.17^†^** | **-0.28^†^** | 0.01 |
| SCD-1 indices |  |  |  |  |  |  |  |  |  |  |  |  |  |  |  |  |
| PLA:PA ratio |  |  |  |  | 1.00 | 0.11 | **0.21**† | 0.02 | **0.20^†^** | 0.03 | **0.49^†^** | **0.56^†^** | **0.30^†^** | **0.48^†^** | **-0.42^†^** | **0.30^†^** |
| OA:SA ratio |  |  |  |  |  | 1.00 | **0.27^†^** | -0.02 | **0.22^†^** | **0.37^†^** | 0.07 | **0.24^†^** | **0.15^†^** | -0.06 | **-0.14^†^** | -0.11 |
| n-3 PUFAs |  |  |  |  |  |  |  |  |  |  |  |  |  |  |  |  |
| α-Linolenic acid (18:3) (ALA) |  |  |  |  |  |  | 1.00 | **0.22^†^** | **0.40^†^** | **0.54^†^** | **0.24^†^** | **0.34^†^** | **0.19^†^** | 0.05 | **-0.28^†^** | **-0.21^†^** |
| Eicosapentanoic acid (20:5) (EPA) |  |  |  |  |  |  |  | 1.00 | **0.71^†^** | **0.23^†^** | **0.25**^†^ | **0.19**^†^ | **0.46^†^** | **0.17**^†^ | **0.19^†^** | **0.31^†^** |
| Docosahexaenoic acid (22:6) (DHA) |  |  |  |  |  |  |  |  | 1.00 | **0.42^†^** | **0.23^†^** | **0.34^†^** | **0.55^†^** | 0.09 | 0.08 | **0.26^†^** |
| n-6 PUFAs |  |  |  |  |  |  |  |  |  |  |  |  |  |  |  |  |
| Linoleic acid (18:2) (LA) |  |  |  |  |  |  |  |  |  | 1.00 | **0.18^†^** | **0.37^†^** | **0.38^†^** | **-0.16^†^** | -0.10 | **-0.31^†^** |
| γ-Linolenic acid (18:3) (GLA) |  |  |  |  |  |  |  |  |  |  | 1.00 | **0.80^†^** | **0.58^†^** | **0.93^†^** | **-0.43^†^** | **0.46^†^** |
| Dihomo-γ-linolenic acid (20:3) (DGLA) |  |  |  |  |  |  |  |  |  |  |  | 1.00 | **0.58^†^** | **0.68^†^** | **-0.65^†^** | **0.34^†^** |
| Arachidonic acid (20:4) (AA) |  |  |  |  |  |  |  |  |  |  |  |  | 1.00 | **0.44^†^** | **0.18**^†^ | **0.72^†^** |
| n-6 PUFA DIs |  |  |  |  |  |  |  |  |  |  |  |  |  |  |  |  |
| GLA:LA ratio (for Δ6 DI) |  |  |  |  |  |  |  |  |  |  |  |  |  | 1.00 | **-0.41**^†^ | **0.58^†^** |
| AA:DGLA ratio (for Δ5 DI) |  |  |  |  |  |  |  |  |  |  |  |  |  |  | 1.00 | **0.24^†^** |
| AA:LA ratio (for total n-6 PUFA DI) |  |  |  |  |  |  |  |  |  |  |  |  |  |  |  | 1.00 |

*Thirty-four subjects who reported regular use of NSAIDs were excluded from the analyses.

†Statistically significant correlations are bolded if P<0.05, and bolded with an † if P<0.01.
