# Supplementary material for: Plasma fatty acids and risk of colon and rectal cancers in the Singapore Chinese Health Study
Source: NPJ Precis Oncol. 2017 Nov 23;1:38. doi: 10.1038/s41698-017-0040-z (PMC5871823; doi:10.1038/s41698-017-0040-z)
Supplement: Supplementary file 2 — Supplementary Table 2 [file 41698_2017_40_MOESM2_ESM.docx]

**Supplementary Table 2.** Spearman correlation coefficients (r) between plasma fatty acids and dietary fatty acids, among control subjects (n=350)

| **Plasma fatty acids** | **Dietary fatty acids** | **r** | ***P* value** |
| --- | --- | --- | --- |
| Palmitic acid (16:0) | Total saturated fatty acids | 0.02 | 0.65 |
| Stearic acid (18:0) | Total saturated fatty acids | <0.01 | 1.00 |
| Palmitoleic acid (16:1) | Total monounsaturated fatty acids | -0.09 | 0.09 |
| Oleic acid (18:1) | Total monounsaturated fatty acids | 0.06 | 0.28 |
| α-Linolenic acid (18:3n-3) | Total n-3 polyunsaturated fatty acids (PUFAs) | 0.13 | 0.02 |
| Eicosapentanoic acid (20:5n-3) | Marine n-3 PUFAs | 0.26 | <0.001 |
| Docosahexaenoic acid (22:6n-3) | Marine n-3 PUFAs | 0.20 | <0.001 |
| Linoleic acid (18:2n-6) | Total n-6 PUFAs | 0.13 | 0.02 |
| γ-Linolenic acid (18:3n-6) | Total n-6 PUFAs | -0.02 | 0.66 |
| Dihomo-γ-linolenic acid (20:3n-6) | Total n-6 PUFAs | -0.03 | 0.64 |
| Arachidonic acid (20:4n-6) | Total n-6 PUFAs | -0.01 | 0.85 |
