# Supplementary material for: Plasma fatty acids and risk of colon and rectal cancers in the Singapore Chinese Health Study
Source: NPJ Precis Oncol. 2017 Nov 23;1:38. doi: 10.1038/s41698-017-0040-z (PMC5871823; doi:10.1038/s41698-017-0040-z)
Supplement: Supplementary file 3 — Supplementary Table 3 [file 41698_2017_40_MOESM3_ESM.docx]

**Supplementary Table 3.** Adjusted odds ratios* and 95% confidence intervals of colorectal cancer by quartile levels of plasma fatty acids and desaturase indices in monounsaturated fatty acid (MUFA) and n-6 polyunsaturated (PUFA) synthesis pathways

|  | 1^st^ (low) | 2^nd^ | 3^rd^ | 4^th^ (high) | *P*_trend_ |
| --- | --- | --- | --- | --- | --- |
| Saturated fatty acids |  |  |  |  |  |
| Palmitic acid (16:0) | 1.00 (Referent) | 0.82 (0.52, 1.3) | 0.89 (0.56, 1.42) | 0.71 (0.43, 1.18) | 0.246 |
| Stearic acid (18:0) | 1.00 (Referent) | 1.07 (0.71, 1.61) | 0.92 (0.60, 1.41) | 0.83 (0.53, 1.30) | 0.343 |
| MUFAs |  |  |  |  |  |
| Palmitoleic acid (16:1) | 1.00 (Referent) | 0.80 (0.52, 1.24) | 0.89 (0.59, 1.35) | 0.73 (0.46, 1.15) | 0.254 |
| Oleic acid (18:1) | 1.00 (Referent) | 0.85 (0.55, 1.30) | 0.92 (0.59, 1.45) | 0.73 (0.45, 1.17) | 0.261 |
| Stearoyl-coenzyme A desaturase (SCD)-1 indices |  |  |  |  |  |
| Palmitoleic:Palmitic ratio | 1.00 (Referent) | 0.86 (0.56, 1.31) | 1.03 (0.66, 1.60) | 0.78 (0.49, 1.23) | 0.439 |
| Oleic:Stearic ratio | 1.00 (Referent) | 1.05 (0.66, 1.66) | 0.87 (0.52, 1.43) | 0.70 (0.4, 1.22) | 0.166 |
| n-3 PUFAs |  |  |  |  |  |
| α-Linolenic acid (18:3) | 1.00 (Referent) | 0.71 (0.46, 1.08) | 0.84 (0.55, 1.29) | 0.73 (0.48, 1.11) | 0.224 |
| Eicosapentanoic acid (20:5) | 1.00 (Referent) | 1.03 (0.67, 1.58) | 0.96 (0.63, 1.47) | 0.87 (0.57, 1.35) | 0.516 |
| Docosahexaenoic acid (22:6) | 1.00 (Referent) | 1.16 (0.75, 1.8) | 1.05 (0.68, 1.62) | 0.93 (0.57, 1.50) | 0.658 |
| n-6 PUFAs |  |  |  |  |  |
| Linoleic acid (18:2) (LA) | 1.00 (Referent) | 0.68 (0.44, 1.05) | 0.80 (0.52, 1.24) | 0.62 (0.38, 0.99) | 0.084 |
| γ-Linolenic acid (18:3) (GLA) | 1.00 (Referent) | 0.90 (0.57, 1.43) | 0.96 (0.62, 1.49) | 0.84 (0.53, 1.31) | 0.509 |
| Dihomo-γ-linolenic (20:3) (DGLA) | 1.00 (Referent) | 0.85 (0.56, 1.28) | 0.90 (0.57, 1.41) | 0.93 (0.59, 1.47) | 0.825 |
| Arachidonic (20:4) (AA) | 1.00 (Referent) | 1.20 (0.77, 1.85) | 2.05 (1.27, 3.32) | 1.50 (0.93, 2.41) | 0.038 |
| n-6 PUFA desaturase indices (DI) |  |  |  |  |  |
| GLA:LA ratio (for Δ6 DI) | 1.00 (Referent) | 1.01 (0.65, 1.57) | 1.13 (0.74, 1.73) | 0.78 (0.49, 1.24) | 0.459 |
| AA:DGLA ratio (for Δ5 DI) | 1.00 (Referent) | 1.05 (0.67, 1.62) | 1.44 (0.94, 2.22) | 1.54 (1.00, 2.36) | 0.024 |
| AA:LA ratio (for total n-6 PUFA DI) | 1.00 (Referent) | 1.82 (1.14, 2.91) | 1.47 (0.92, 2.36) | 2.06 (1.28, 3.31) | 0.012 |

*Odds ratios are adjusted for body mass index (<20, 20-24, 24-28, ≥28 kg/m^2^), smoking (never, light, heavy), education level (none, primary, ≥secondary), alcohol use (none, <7, ≥7 drinks/wk), weekly physical activity (yes, no), history of diabetes (yes, no).
