# Supplementary material for: Plasma fatty acids and risk of colon and rectal cancers in the Singapore Chinese Health Study
Source: NPJ Precis Oncol. 2017 Nov 23;1:38. doi: 10.1038/s41698-017-0040-z (PMC5871823; doi:10.1038/s41698-017-0040-z)
Supplement: Supplementary file 4 — Supplementary Table 4 [file 41698_2017_40_MOESM4_ESM.docx]

**Supplmental Table 4.** Adjusted odds ratios ^a^ and 95% confidence intervals of colon cancer by quartile levels of plasma palmitic acid (16:0), oleic acid (18:1), a-Linolenic acid (18:3), and AA:DGLA, a desaturase indice in n-6 polyunsaturated fatty acid (PUFA) synthesis pathway, stratified by fasting status

| Fatty acid | Fasting status | 1^st^ (low) | 2^nd^ | 3^rd^ | 4^th^ (high) | *P*_trend_ | *P* _interaction_ |
| --- | --- | --- | --- | --- | --- | --- | --- |
|  |  |  |  |  |  |  |  |
| Palmitic acid (16:0) | **Nonfasting** |  |  |  |  |  | 0.189 |
|  | Controls/Cases | 38/50 | 35/35 | 46/47 | 55/39 |  |  |
|  | OR (95%CI) | 1.00 (Referent) | 0.75 (0.39-1.43) | 0.73 (0.40-1.32) | 0.48 (0.26-0.87) | 0.021 |  |
|  | **Fasting** |  |  |  |  |  |  |
|  | Controls/Cases | 15/14 | 12/12 | 5/9 | 5/5 |  |  |
|  | OR (95%CI) | 1.00 (Referent) | 1.05 (0.31-3.56) | 1.30 (0.30-5.69) | 0.75 (0.15-3.69) | 0.886 |  |
| Oleic acid (18:1) | **Nonfasting** |  |  |  |  |  | 0.327 |
|  | Controls/Cases | 38/52 | 41/38 | 43/44 | 52/37 |  |  |
|  | OR (95%CI) | 1.00 (Referent) | 0.61 (0.33-1.15) | 0.67 (0.36-1.24) | 0.44 (0.24-0.81) | 0.016 |  |
|  | **Fasting** |  |  |  |  |  |  |
|  | Controls/Cases | 13/17 | 12/7 | 7/11 | 5/5 |  |  |
|  | OR (95%CI) | 1.00 (Referent) | 0.51 (0.14-1.88) | 0.87 (0.23-3.24) | 0.56 (0.12-2.69) | 0.549 |  |
| α-Linolenic acid (18:3) | **Nonfasting** |  |  |  |  |  | 0.886 |
|  | Controls/Cases | 34/55 | 37/33 | 50/47 | 53/36 |  |  |
|  | OR (95%CI) | 1.00 (Referent) | 0.54 (0.28-1.03) | 0.57 (0.31-1.03) | 0.40 (0.21-0.74) | 0.006 |  |
|  | **Fasting** |  |  |  |  |  |  |
|  | Controls/Cases | 13/19 | 12/9 | 8/10 | 4/2 |  |  |
|  | OR (95%CI) | 1.00 (Referent) | 0.27 (0.07-1.07) | 0.61 (0.16-2.31) | 0.10 (0.01-1.14) | 0.131 |  |
| AA:DGLA ratio (for Δ5 DI) | **Nonfasting** |  |  |  |  |  | 0.228 |
|  | Controls/Cases | 55/31 | 47/36 | 37/55 | 35/49 |  |  |
|  | OR (95%CI) | 1.00 (Referent) | 1.37 (0.72-2.59) | 3.08 (1.64-5.77) | 2.83 (1.48-5.39) | <0.001 |  |
|  | **Fasting** |  |  |  |  |  |  |
|  | Controls/Cases | 10/10 | 6/4 | 10/13 | 11/13 |  |  |
|  | OR (95%CI) | 1.00 (Referent) | 0.74 (0.13-4.35) | 1.05 (0.26-4.19) | 1.01 (0.27-3.74) | 0.899 |  |

^a^ Odds ratios are adjusted for body mass index (<20, 20-24, 24-28, ≥28 kg/m^2^), smoking (never, light, heavy), education level (none, primary, ≥secondary), alcohol use (none, <7, ≥7 drinks/wk), weekly physical activity (yes, no), history of diabetes (yes, no).
