# Supplementary material for: Plasma fatty acids and risk of colon and rectal cancers in the Singapore Chinese Health Study
Source: NPJ Precis Oncol. 2017 Nov 23;1:38. doi: 10.1038/s41698-017-0040-z (PMC5871823; doi:10.1038/s41698-017-0040-z)
Supplement: Supplementary file 5 — Supplementary Table 5 [file 41698_2017_40_MOESM5_ESM.docx]

**Supplementary Table 5.** Adjusted odds ratios (OR)* and 95% confidence intervals (CI) of colon cancer comparing highest to lowest quartile of selected plasma fatty acids and desaturase indices excluding cases identified during the first 2 and 4 years of follow-up and their matched controls.

|  | First 2 year excluded  (n = 141 cases and 141 controls) | |  | First 4 years excluded  (n = 90 cases and 90 controls) | |
| --- | --- | --- | --- | --- | --- |
|  | OR (95% CI) | *P_trend_* |  | OR (95% CI) | *P_trend_* |
| Oleic acid (18:1) | 0.57 (0.25, 1.31) | 0.27 |  | 0.61 (0.20, 1.9) | 0.60 |
| Oleic:Stearic acid ratio (18:1/18:0) | 0.27 (0.09, 0.78) | 0.02 |  | 0.48 (0.14, 1.65) | 0.28 |
| α-Linolenic acid (18:3) | 0.65 (0.32, 1.33) | 0.25 |  | 0.56 (0.21, 1.49) | 0.12 |
| Linoleic acid (18:2) | 0.72 (0.33, 1.58) | 0.30 |  | 0.49 (0.14, 1.65) | 0.20 |
| Arachidonic acid (20:4) | 1.73 (0.83, 3.60) | 0.10 |  | 3.96 (1.40, 11.21) | <0.01 |
| Arachidonic:Linoleic acid ratio (20:4/18:2) | 3.08 (1.36, 7.00) | 0.10 |  | 11.97 (2.93, 48.87) | 0.01 |

*Odds ratios are adjusted for body mass index (<20, 20-24, 24-28, ≥28 kg/m^2^), smoking (never, light, heavy), education level (none, primary, ≥secondary), alcohol use (none, <7, ≥7 drinks/wk), weekly physical activity (yes, no), history of diabetes (yes, no).
