# Supplementary material for: Plasma fatty acids and risk of colon and rectal cancers in the Singapore Chinese Health Study
Source: NPJ Precis Oncol. 2017 Nov 23;1:38. doi: 10.1038/s41698-017-0040-z (PMC5871823; doi:10.1038/s41698-017-0040-z)
Supplement: Supplementary file 6 — Supplementary Table 6 [file 41698_2017_40_MOESM6_ESM.docx]

**Supplementary Table 6.** Adjusted odds ratios (OR)* and 95% confidence intervals (CI) of colon cancer comparing highest to lowest quartile of selected plasma fatty acids and desaturase indices excluding cases according to median follow-up time.

|  | < 3 years  (n = 97 cases and 97 controls) | |  | ≥ 3 years  (n = 114 cases and 114 controls) | | *P_heterogeneity_* |
| --- | --- | --- | --- | --- | --- | --- |
|  | OR (95% CI) | *P_trend_* |  | OR (95% CI) | *P_trend_* |  |
| Oleic acid (18:1) | 0.37 (0.14, 0.98) | 0.09 |  | 0.42 (0.16, 1.09) | 0.13 | 0.43 |
| Oleic:Stearic acid ratio (18:1/18:0) | 0.52 (0.17, 1.58) | 0.26 |  | 0.31 (0.09, 1.00) | 0.04 | 0.26 |
| α-Linolenic acid (18:3) | 0.38 (0.16, 0.91) | 0.048 |  | 0.40 (0.17, 0.92) | 0.04 | 0.47 |
| Linoleic acid (18:2) | 0.34 (0.14, 0.86) | 0.03 |  | 0.55 (0.21, 1.43) | 0.14 | 0.24 |
| Arachidonic acid (20:4) | 0.65 (0.24, 1.81) | 0.50 |  | 2.25 (1.00, 5.04) | 0.03 | 0.03 |
| Arachidonic:Linoleic acid ratio (20:4/18:2) | 3.02 (1.09, 8.32) | 0.07 |  | 4.49 (1.73, 11.68) | 0.07 | 0.29 |

*Odds ratios are adjusted for body mass index (<20, 20-24, 24-28, ≥28 kg/m^2^), smoking (never, light, heavy), education level (none, primary, ≥secondary), alcohol use (none, <7, ≥7 drinks/wk), weekly physical activity (yes, no), history of diabetes (yes, no).
