# Supplementary material for: Plasma fatty acids and risk of colon and rectal cancers in the Singapore Chinese Health Study
Source: NPJ Precis Oncol. 2017 Nov 23;1:38. doi: 10.1038/s41698-017-0040-z (PMC5871823; doi:10.1038/s41698-017-0040-z)
Supplement: Supplementary file 7 — Supplementary Table 7 [file 41698_2017_40_MOESM7_ESM.docx]

**Supplementary Table 7.** Number of colon and rectal cancer cases and their matched controls by quartile levels of plasma fatty acids and their desaturase indices

|  | 1^st^ (low) | 2^nd^ | 3^rd^ | 4^th^ (high) |
| --- | --- | --- | --- | --- |
| **Monounsaturated fatty acid (MUFA) synthesis pathway** |  |  |  |  |
| Saturated fatty acids |  |  |  |  |
| Palmitic acid (16:0), µmol/dL | <214.0 | 214.0-265.7 | 265.8-345.4 | >345.4 |
| Colon, cases/controls | 64/53 | 47/47 | 56/51 | 44/60 |
| Rectal, cases/controls | 32/35 | 36/40 | 38/37 | 33/27 |
| Stearic acid (18:0), µmol/dL | <78.3 | 78.3-93.3 | 93.4-116.5 | >116.5 |
| Colon, cases/controls | 61/51 | 54/51 | 54/50 | 42/59 |
| Rectal, cases/controls | 31/37 | 43/36 | 30/38 | 35/28 |
| MUFAs |  |  |  |  |
| Palmitoleic acid (16:1), µmol/dL | <13.1 | 13.1-19.5 | 19.6-31.2 | >31.2 |
| Colon, cases/controls | 62/54 | 51/51 | 56/50 | 42/56 |
| Rectal, cases/controls | 39/34 | 29/36 | 37/38 | 34/31 |
| Oleic acid (18:1), µmol/dL |  |  |  |  |
| Colon, cases/controls | 69/51 | 45/53 | 55/50 | 42/57 |
| Rectal, cases/controls | 27/37 | 39/34 | 39/38 | 34/30 |
| Stearoyl-coenzyme A desaturase (SCD)-1 indices |  |  |  |  |
| Palmitoleic:Palmitic acid ratio | <0.055 | 0.055-0.072 | 0.073-0.102 | >0.102 |
| Colon, cases/controls | 62/57 | 49/49 | 59/53 | 41/52 |
| Rectal, cases/controls | 35/31 | 31/38 | 38/35 | 35/35 |
| Oleic:Stearic acid ratio | <3.1 | 3.1-3.6 | 3.7-4.3 | >4.3 |
| Colon, cases/controls | 58/52 | 61/55 | 48/48 | 43/55 |
| Rectal, cases/controls | 30/35 | 35/32 | 39/39 | 33/31 |
|  |  |  |  |  |
| **n-3 Polyunsaturated fatty acids (PUFAs)** |  |  |  |  |
| α-Linolenic acid (18:3), µmol/dL | <1.9 | 1.9-2.6 | 2.7-3.8 | >3.8 |
| Colon, cases/controls | 74/47 | 42/49 | 57/58 | 38/57 |
| Rectal, cases/controls | 33/41 | 33/38 | 34/30 | 39/30 |

**Supplementary Table 7** (continued)

|  | 1^st^ (low) | 2^nd^ | 3^rd^ | 4^th^ (high) |
| --- | --- | --- | --- | --- |
| Eicosapentanoic acid (20:5), µmol/dL | <2.4 | 2.4-3.4 | 3.5-5.1 | >5.1 |
| Colon, cases/controls | 57/57 | 50/51 | 56/48 | 48/55 |
| Rectal, cases/controls | 33/31 | 43/36 | 30/40 | 33/32 |
| Docosahexaenoic acid (22:6), µmol/dL | <17.6 | 17.6-24.9 | 25.0-35.2 | >35.2 |
| Colon, cases/controls | 51/61 | 60/51 | 50/45 | 50/54 |
| Rectal, cases/controls | 32/27 | 36/36 | 38/43 | 33/33 |
| **n-6 PUFA synthesis pathway** |  |  |  |  |
| n-6 PUFAs |  |  |  |  |
| Linoleic acid (18:2) (LA), µmol/dL | <498.1 | 498.1-585.7 | 585.8-716.0 | >716.0 |
| Colon, cases/controls | 73/48 | 45/48 | 49/59 | 44/56 |
| Rectal, cases/controls | 40/40 | 30/39 | 39/29 | 30/31 |
| γ-Linolenic acid (18:3) (GLA), µmol/dL | <1.04 | 1.04-1.67 | 1.68-2.88 | >2.88 |
| Colon, cases/controls | 58/42 | 51/57 | 58/56 | 44/56 |
| Rectal, cases/controls | 35/46 | 34/30 | 36/32 | 34/31 |
| Dihomo-γ-linolenic acid (20:3) (DGLA), µmol/dL | <5.53 | 5.53-7.37 | 7.38-10.31 | >10.31 |
| Colon, cases/controls | 55/47 | 54/51 | 58/56 | 44/57 |
| Rectal, cases/controls | 39/41 | 26/36 | 28/32 | 46/30 |
| Arachidonic acid (20:4) (AA), µmol/dL | <54.3 | 54.4-69.1 | 69.2-92.2 | >92.2 |
| Colon, cases/controls | 41/56 | 50/50 | 69/55 | 51/50 |
| Rectal, cases/controls | 26/32 | 28/37 | 47/33 | 38/37 |
| n-6 PUFA desaturase indices (DI) |  |  |  |  |
| GLA:LA ratio (for Δ6 DI) (x 1,000) | <1.64 | 1.64-2.86 | 2.87-4.88 | >4.88 |
| Colon, cases/controls | 49/45 | 57/55 | 66/52 | 39/59 |
| Rectal, cases/controls | 38/43 | 32/32 | 37/36 | 32/28 |
| AA:DGLA ratio (for Δ5 DI) | <6.74 | 6.74-9.20 | 9.21-12.05 | >12.05 |
| Colon, cases/controls | 41/65 | 40/53 | 68/47 | 62/46 |
| Rectal, cases/controls | 32/23 | 31/34 | 33/41 | 43/41 |
| AA:LA ratio (for total n-6 PUFA DI) | <0.092 | 0.092-0.121 | 0.122-0.155 | >0.155 |
| Colon, cases/controls | 30/55 | 69/53 | 41/59 | 71/44 |
| Rectal, cases/controls | 27/33 | 30/34 | 42/29 | 40/43 |
